# The need for ventilators in the developing world: An opportunity to improve care and save lives

**DOI:** 10.7189/jogh.04.010303

**Published:** 2014-06

**Authors:** Vijay Krishnamoorthy, Monica S. Vavilala, Charles N. Mock

**Affiliations:** 1 Department of Anesthesiology & Pain Medicine, University of Washington, Seattle, WA, USA; 2 Harborview Injury Prevention and Research Center, Washington, Seattle, WA, USA; 3 Departments of Pediatrics, Neurological Surgery and Radiology, University of Washington, Seattle, WA, USA; 4 Departments of Surgery and Epidemiology, University of Washington, Seattle, WA, USA

The drive to breathe is a fundamental human and biologic behavior, regulated by a complex system of checks and balances in the body. When respiratory mechanics are deregulated by injury, infection, coma, or a host of other conditions, the biologic equilibrium shifts into a state of respiratory failure. When this occurs, mechanical ventilation can be a life saving therapy. While commonplace in developed countries, critical care is at its infancy in many developing countries [1], where basic technology is often not available. Thus, while many lives are saved in developed nations through the provision of mechanical ventilation, patients in many developing nations often die from otherwise reversible causes due to lack of resources, education, and training.

In this viewpoint paper, we will explore arguments in support of and against the provision of one vital resource – mechanical ventilators – in resource–poor settings. Furthermore, we will address both the benefits and challenges in implementing a program of increased provision of mechanical ventilators. Lastly, we will provide some solutions to address potential barriers to this initiative.

## BURDEN OF RESPIRATORY FAILURE IN DEVELOPING COUNTRIES: A CHALLENGE

Much of our data about the burden of respiratory failure worldwide comes primarily from developed nations. Unfortunately, because the disparity in quality of care within developing countries is wide [2], no reliable comparative epidemiological data of critical illness syndromes, such as acute lung injury and sepsis, are available. While respiratory failure may be fairly easy to diagnose clinically (such as hypoxia or increased work of breathing), it is a consequence of a primary disease process (ie, pneumonia) – thus, as a secondary process, collection of epidemiologic data are challenging in resource–poor settings. This results in the comparative epidemiology (between resource–intensive and resource–poor settings) of critical illness and respiratory failure being heterogeneous [3,4]. Furthermore, mortality after critical illness is related to both clinical decisions to limit intensive care and the consequences of the disease; therefore, countries with the resources to provide intensive care for patients with comorbid illnesses will have a perceived higher burden of critical illness associated with these disorders compared to countries which do not initiate treatment in the first place.

With a potentially high burden and mortality of respiratory failure in developing nations, the provision of mechanical ventilators may help save lives if implemented in a thoughtful fashion. Thus, good outcomes in this patient population may contribute to healthier patients with better future productivity and economic potential. Despite this, several barriers to implementing a greater number of ventilators exist, including perceived high cost, the need for education, and a lack of research in ventilator protocols for resource–poor settings.

## BENEFITS OF THE PROVISION OF VENTILATORS IN DEVELOPING COUNTRIES

As previously mentioned, the epidemiologic data regarding the burden of respiratory failure in developing countries is poor and may potentially be underrepresented due to large proportion of uncaptured data in patients in whom intensive care was never initiated in the first place due to perceived futility of treatment. Although classically thought to only benefit a small segment of patients, mechanical ventilation actually can help a wide variety of patients including patients with injury, non–communicable diseases (NCDs), and communicable diseases such as the human immunodeficiency virus (HIV) and malaria. For example, while it has been recognized that NCDs are beginning to account for a larger burden of disease in developing countries [5], decompensated NCDs (ie, heart failure exacerbations) commonly require critical care and mechanical ventilation. In the same vein, a patient with HIV infection may also decompensate from the acquisition of opportunistic infections and require mechanical ventilation.

Youth are often are disproportionately affected by critical illness and respiratory failure in the developing world; thus, a large amount of patients who have many years of contribution to society needlessly die due primarily to a lack of resources and education [6]. Furthermore, the limited data comparing critical care in Europe versus developing nations confirms that patients in developing countries tended to be younger and had an improved prior health status [7,8]; thus, the potential for recovery and productivity exists. An example of this situation is care for young patients with traumatic brain injury (TBI). In developed nations, TBI outcomes have significantly improved through careful adherence to the Brain Trauma Foundation guidelines, which emphasize appropriate respiratory care and oxygenation of the brain–injured patient [9]. In developing countries, unfortunately, many of these young patients are not given a chance for survival because of the lack of basic ventilators for respiratory support.

While mechanical ventilation can be viewed as a prolonged task in some patients, the majority of patients would only require a short course of mechanical ventilation. This is because the four most common admission criteria requiring ventilation in intensive care units in developing countries are postsurgical treatment, infectious diseases, trauma, and peripartum maternal or neonatal complications [10] – the majority of these processes are reversible over a short period of time. Therefore, the provision of a short duration of mechanical ventilation has the potential to help patients with a variety of reversible pathologies.

## ARGUMENTS AGAINST THE PROVISION OF VENTILATORS IN DEVELOPING COUNTRIES

A primary argument against the provision of ventilators in developing nations is centered on the increased cost of the intervention. While tackling prevention, communicable diseases, and NCDs, the strain that providing ventilators puts on funding agencies can be substantial. Furthermore, at a very high cost per ventilator even in developed countries (average anywhere from US$ 20 000 up to US$ 100 000), ventilators by no means are a cheap intervention. To address these issues, basic ventilators for developing countries are being developed at much lower costs. While the capabilities of these machines are not nearly as robust as more expensive machines used in developed countries, the vast majority of patients even in developed countries are ventilated for a short duration and require the “minimal settings” that most ventilators can provide. In addition, the majority evidence–based maneuvers do not require complex ventilation strategies (ie, lung–protective ventilation) [11], and can be provided with a basic ventilator. Lastly, as mentioned above, funding priorities can continue to be met, as ventilators will improve care for patients with diseases under well–funded projects (ie, decompensated HIV, malaria, NCDs). Even if funding for basic ventilators is provided, it will be a disproportionately small amount of funding as compared to other disease states such as HIV [12].

While we have explored the cost to society and funding agencies as a barrier to implementing mechanical ventilation in resource–poor settings, a likely important reason for ceasing (or, not even starting) intensive care in developing countries is the family’s inability to keep up with the cost of caring for the patient – in the extreme case, sometimes driving families into poverty. On the other hand, if cost to the family was not an issue (as is the case in many developed countries), the challenge may shift to clinical ethics; because of religious or cultural beliefs coupled with a misunderstanding of treatment effectiveness (a situation that is often faced in developed countries as well), patients receive mechanical ventilation long after it will be of any benefit. Therefore, an ethical framework would be necessary to advise both doctors and patients of possible decisions on the withdrawal of care or transition to “comfort” care. Furthermore, if demand outstrips the supply of ventilators, decision rules will need to be put into place to ethically select which patients with benefit the most from the therapy.

Aside from cost, another compelling argument against the provision of ventilators in resource–poor settings is the inadequacy of current systems to appropriately care for patients on ventilators and the ventilators themselves [13]. The initial of care for the patient with respiratory failure (ie, from trauma) is often in the field, and appropriate emergency medical services (EMS) training must involve appropriate initial care and triage of these patients.

It must be understood that mechanical ventilation is a complex task more than just merely “turning on” the machine. The act of putting a patient on mechanical ventilation requires the provision of an endotracheal tube (or tightly–fitting non–invasive face mask), making adjustments to the machine to meet patient needs, responding to ventilator crises, adequate sedation of the patient, and appropriate patient weaning and eventual liberation of the patient from mechanical ventilation. Second, ventilators can be vulnerable machines and require appropriate maintenance. Third, ventilators require both electricity and compressed oxygen, both potentially scarce resources in developing countries; in order to fulfill the ethic principle of equity, basic oxygen and electricity must be available throughout a region before considering the institution of mechanical ventilation. Thus, it is apparent that beyond simply providing ventilators to resource–poor settings, appropriate systems must be put into place to address issues of both care of the ventilated patient and care of the ventilator itself; the opportunity cost of this may involve shifting resources from other public health priorities, thus system changes need to be implemented in a thoughtful, evidence–based manner.

## OVERCOMING BARRIERS TO THE PROVISION OF MECHANICAL VENTILATION

Reaching to goal of delivering high quality respiratory care is lofty, but very possible with a systematic approach to funding, education, and research. First and foremost, educational initiatives would be needed to address several issues; not only would physicians and nurses need training on appropriate care of the ventilated patient, but staff would also need to be trained on the care and maintenance of these machines. Second, systems would need to be in place to create protocols for complex processes to provide consistent evidence–based care to patients – checklists have been proven very successful in this regard. Third, several protocols from developed countries will likely need to be modified to best meet the needs and resources of developing countries [14]. Fourth, government systems would need to be in place to assure consistent power (ie, electricity) and oxygen for the machines. Fifth, a triage system would likely be needed to “regionalize” care for sick and complex patients who require more advanced therapies beyond basic ventilator management. Sixth, from a donor and funding perspective, greater education needs to be provided to donors that critical care and mechanical ventilation can be cost–effective, and that most evidence–based critical care interventions tend to be inexpensive [1]. Furthermore, the economic implications and advantages of decreasing mortality in young populations would need to be stressed. Lastly, research should be performed in resource–poor settings to focus on needs assessment, education, implementation, and cost–effectiveness. While the necessary steps above seem complex, we have already proven that the global health community can tackle complex obstacles – the successful implementation of HIV care (one of the most complex diseases known to modern society) in some of the world’s most destitute regions is proof of this.

**Figure:**
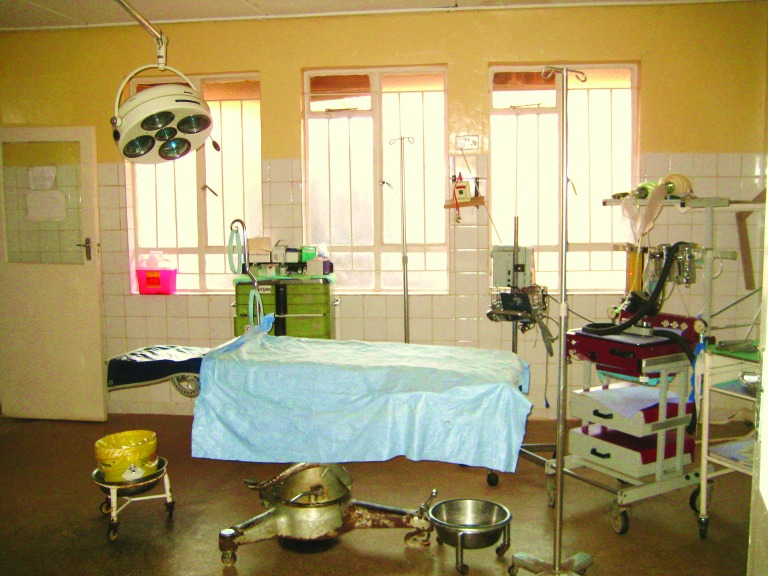
Photo: Courtesy of Alasdair Campbell, personal collection

## CONCLUSION

The first formal use of ventilators in modern medicine is reported to have started by Dr Bjørn Ibsen in Copenhagen in 1953, whose use of mechanical ventilation helped to save scores of lives of patients with polio who were dying of respiratory failure, reducing mortality from 87% to 25% [15]. Over the subsequent decades, the use of mechanical ventilation in developed countries has been refined, and now is one of the defining interventions of critical care medicine in the developed world. Through extensive experience, creation of effective systems, protocol development, and research, mechanical ventilation has become a life–saving intervention. Similar to Copenhagen in 1953, in present–day resource–poor settings, the practice of intensive care is likewise in an early stage of development, and the provision of ventilators may have the potential to have a positive impact on reducing mortality from a myriad of etiologies. As with any new intervention, providing ventilators to developing nations comes with not only benefits, but also a host of new challenges to overcome. The key to moving forward is to help funding agencies understand the benefits, while putting together a detailed plan (as outlined above) to address the limitations. With this understanding, the provision of mechanical ventilators to developing countries has the unique potential to help make a dramatic improvement in the care of the world’s most vulnerable patients.
